# Immune mechanisms in cerebral ischemic tolerance

**DOI:** 10.3389/fnins.2014.00044

**Published:** 2014-03-04

**Authors:** Lidia Garcia-Bonilla, Corinne Benakis, Jamie Moore, Costantino Iadecola, Josef Anrather

**Affiliations:** Brain and Mind Research Institute, Weill Cornell Medical CollegeNew York, NY, USA

**Keywords:** preconditioning, ischemic tolerance, stroke, TLR, epigenetics, microRNAs, TNF, inflammation

## Abstract

Stressor-induced tolerance is a central mechanism in the response of bacteria, plants, and animals to potentially harmful environmental challenges. This response is characterized by immediate changes in cellular metabolism and by the delayed transcriptional activation or inhibition of genetic programs that are not generally stressor specific (cross-tolerance). These programs are aimed at countering the deleterious effects of the stressor. While induction of this response (preconditioning) can be established at the cellular level, activation of systemic networks is essential for the protection to occur throughout the organs of the body. This is best signified by the phenomenon of remote ischemic preconditioning, whereby application of ischemic stress to one tissue or organ induces ischemic tolerance (IT) in remote organs through humoral, cellular and neural signaling. The immune system is an essential component in cerebral IT acting simultaneously both as mediator and target. This dichotomy is based on the fact that activation of inflammatory pathways is necessary to establish IT and that IT can be, in part, attributed to a subdued immune activation after index ischemia. Here we describe the components of the immune system required for induction of IT and review the mechanisms by which a reprogrammed immune response contributes to the neuroprotection observed after preconditioning. Learning how local and systemic immune factors participate in endogenous neuroprotection could lead to the development of new stroke therapies.

## Introduction

Inflammation is a central component in the pathophysiology of cerebral ischemia. Brain ischemia triggers both a local and systemic inflammatory response. These responses play contradictory roles: contributing to progression of the ischemic lesion on one hand, and to processes of tissue repair in the injured brain on the other (Iadecola and Anrather, 2011a; Macrez et al., 2011). Since post-ischemic inflammation shows detrimental and beneficial aspects, anti-inflammatory therapies that indiscriminately target both arms of this immune response have not been successful (Iadecola and Anrather, 2011a; Macrez et al., 2011). Therefore, a deeper understanding of post-ischemic inflammation is needed in order to harness its beneficial effects for therapeutic purposes.

In addition to its beneficial role in the repair and regeneration of ischemic tissue, inflammatory pathways are also involved in evoking neuroprotective mechanisms that lead to ischemic tolerance (IT). The brain is equipped with a remarkable capacity to mount self-protective programs that are tuned to limit the deleterious effects of ischemia, commonly referred to as endogenous neuroprotection (Iadecola and Anrather, 2011b). These protective programs can be evoked by preconditioning (PC) stimuli (Table 1), such as sublethal stressors, resulting in IT (Kirino, 2002; Narayanan et al., 2013). Thus, understanding the endogenous inflammatory pathways involved in brain IT may lead to novel therapeutic strategies for the prevention and repair of neuronal damage in stroke patients (Shpargel et al., 2008; Macrez et al., 2011).

**Table 1:** **List of preconditioning stimuli inducing ischemic tolerance**.

**Abbreviation**	**Preconditioning stimulus**	**Achieved by**
IPC	Ischemic preconditioning	Transient focal and global cerebral ischemia
RIPC	Remote ischemic preconditioning	Transient occlusion of femoral arteries, mesenteric artery or renal artery
HPC	Hypoxic preconditioning	Exposure to a hypoxic gas mixture
HBO-PC	Hyperoxia or hyperbaric oxygen preconditioning	Exposure to hyperoxia (high oxygen tension) or hyperbaricity (high atmospheric pressure)
Hypo- or hyper-thermic PC	Hypothermic or hyperthermic preconditioning	Decrease or increase of body temperature, respectively
Anesthetic PC	Anesthetic preconditioning	Inhalation of low dose isoflurane or halothane
CSD-PC	Cortical spreading depression preconditioning	Propagation of depolarization wave across the cortical surface
Seizure PC	Seizure preconditioning	Kainic acid injections that induce mild epileptic activity
Exercise PC	Exercise preconditioning	Motor training (treadmill)
TLR-PC	Toll-like receptor preconditioning	Administration of low dose of TLR ligands
LPS-PC	Lipopolysaccharide preconditioning	Administration of low dose of bacterial lipopolysaccharide endotoxin

Cerebral IT can be acquired by several stimuli including short episodes of transient focal and global cerebral ischemia, remote organ ischemia, hypoxia, hypothermia, hyperthermia, exposure to inhalation anesthetics, cortical spreading depression (CSD), brief episodes of seizures or by exposure to low-dose bacterial lipopolysaccharide (LPS) prior to cerebral ischemia (Gidday, 2006; Kunz et al., 2007; Shpargel et al., 2008). IT is characterized by the activation of evolutionary conserved programs that serve to increase the resistance of the brain to ischemia. In the case of early preconditioning, these programs can be activated through preexisting signaling modules that converge in the mitochondria to improved mitochondrial function and energy metabolism during the ischemic event (Dirnagl and Meisel, 2008). In contrast, delayed PC involves gene transcription and protein synthesis (Kitagawa et al., 1990). Because activation of transcription is the main mechanism by which many pro-inflammatory signaling cascades induce a specific cellular response, inflammation is thought to be a significant component of delayed PC. Table 2 summarizes the inflammatory pathways involved in IT achieved by different PC stimuli (Table 1). Inflammation is activated by innate immune receptors such as Toll-like receptors (TLRs) or cytokines receptors such as tumor necrosis factor receptor 1 (TNFR1) and interleukin-1 receptor (IL-1R). Activation of these pathways results in the induction of inflammatory genes that are mediators or effectors of the PC stimulus. In addition to conferring cytoprotection directly, the acquired IT protects the brain by suppressing post-ischemic proinflammatory gene expression, microglial and endothelial activation and leukocyte infiltration (Huang et al., 2006; Iadecola and Anrather, 2011b).

**Table 2:** **Molecular inflammatory mechanisms of brain preconditioning**.

**PC stimulus**	**PC type**	**Receptors**	**Transducers and effectors induced by PC**	**References**
Ischemia	BCCAo	TLR4	NF-κB, TNF-α, iNOS, COX-2 (48 h)	Pradillo et al., 2009
	MCAo	TNFR1	TACE/TNF-α, NF-κB (48 h)	Pradillo et al., 2005
			TNF-α/TACE (48 h)	Cárdenas et al., 2002
			HO-1, COX-2/PGE_2_/ PI3K/Akt (24 h)	Park et al., 2008
			IL-1β gene (6 h), IL-1ra (6 and 24 h)	Shin et al., 2009
			IL-1β (6 h)	Wang et al., 2000
	Remote forearm ischemia	TLR4, TNFR6	HSP70, Calpastatin, TIMP1, ↓caspase-8; PI3KCA, SNAP-23 (24 h)	Konstantinov et al., 2004
			↓neutrophil adhesion and phagocytosis, IL–1β, IL-10 (24 h and 10 days)	Shimizu et al., 2010
			↓platelet activation (PMAs) (5 and 45 min)	Pedersen et al., 2011
	Remote femoral artery ischemia		↑reperfusion (possible protection of endothelium) (48 h)	Vlasov et al., 2005
Hypoxia	8% oxygen, 20 min to 4 h		TNF-α, ceramide (24 h)	Liu et al., 2000
			SphK/S1P (2–4 h), HIF, SphK2, CCL2 (12–24 h)	Wacker et al., 2012
			neuronal CCL2 (12 h), endothelial CCL2 (2 days)	Stowe et al., 2012
			PI3K/Akt/GSK-3β, NF-κB (1–24 h) (post-hypoxic ischemia)	Yin et al., 2007
HBO-PC	Hyperbaric		COX-2 (1–3 days) (post-global ischemia)	Cheng et al., 2011
			HIF-1α, EPO (12 h) (post-MCAo)	Peng et al., 2008
	Normobaric hyperoxia		TNF-α, TACE (24 h)	Bigdeli and Khoshbaten, 2008
Hypothermia	33.0°C, 4 h of reperfusion		↓PMN leucocytes, intercellular adhesion molecule-1 mRNA (4–22 h) (post-MCAo)	Kawai et al., 2000
Hyperthermia	41.5–42°C, 15 min		↑Cortisol (possible prevention of BBB disruption) (24 h)	Ikeda et al., 1999
	38 or 40°C, 6 h		↑HIF-1 alpha expression, HIF-1 binding activity (astrocytes) (0 h)	Du et al., 2010
	42–43°C, 2 h		↑HSP70 (glial, endothelial cells) (24 h)	Ota et al., 2000
Anesthetics	Isoflurane or halothane		↑iNOS (6–24 h)	Kapinya et al., 2002; Zhao and Zuo, 2004
			↑HO-1, NO and TNF-α (macrophages) (24 h)	Li et al., 2009
CSD	0.5 M KCl		↑ERK and COX-2 expression (0–8 h and 2–3 days)	Horiguchi et al., 2005, 2006
	3 M KCl	NMDA receptor	↑TNF-α, IL–1β (4 h)	Jander et al., 2001
Seizure	Kainic acid, bicuculline methiodide, or electrical stimulation		Unknown	Vezzani et al., 2002
Exercise	Treadmill		↑TNF-α (0 h)	Ding et al., 2005
TLR ligands	LPS	TLR4	NF-κB suppression; IRF3, Ship1, Tollip, p105 (post-MCAo) (72 h)	Sly et al., 2004; Vartanian et al., 2011
			Genes related to TLR pathway and cytokine–cytokine receptor interaction pathway (3 h)	Marsh et al., 2009b; Stevens et al., 2011
			IRF3, IFNβ (3 and 24 h)	Marsh et al., 2009b
			↑Ceramide (6–12 and 48 h)	Zimmermann et al., 2001
			↑PMN infiltration (post-MCAo) (6 and 24 h)	Ahmed et al., 2000
	CpG ODN	TLR9	IRF3, IRF7, type I IFN gene expression (post-MCAo) (3 and 24 h); TNF-α (serum) (1 h)	Stevens et al., 2008, 2011
	GDQ	TLR7, IFNAR	IRF7, IFNα (1–2 h)	Leung et al., 2012
	Poly-ICLC	TLR3	↑plasma levels of IL-1β, IL-6, IL-12, TNF-α, and IFNγ compared to LPS-PC (3 h)	Packard et al., 2012
			↑IFN-β (protein 6–8 h and mRNA 6–24 h), preservation of BBB endothelial cell	Gesuete et al., 2012
	Pam3CSK4	TLR2	↑zonula occludens-1 (ZO-1), no loss of occludin protein inducing preservation of BBB (6 and 24 h)	Hua et al., 2008

*A PC stimulus (column 1) may be divided in several PC types (column 2) that through the stimulation of several receptors (column 3), activate different transducers and effectors of inflammatory pathways (column 4), accounting for the protective mechanism of the brain. Time points in brackets indicate the time point of analysis relative to the PC induction; post-MCAo indicates that the analysis was performed after induction of index ischemia*.

*ATA, atmosphere abolute pressure; BBB, Blood Brain Barrier; BCCAo, Bilateral Common Carotid Artery Occlusion; CCL2, chemokine (C-C motif) ligand; CCR1, 5, Chemokine Receptor 1, 5; COX-2, Cyclooxygenase 2; CpG ODN, cytosine-guanine oligodeoxynucleotides; CSD, Cortical Spreading Depression, EPO, Erythropoietin; GDQ, Gardiquimod; GSK-3β, Glycogen Synthase Kinase 3; HBO-PC, Hyperbaric Oxygen Preconditioning; HIF, Hypoxia-Inducible Factor; HO-1, Heme Oxygenase-1; HSP70, Heat Shock Protein 70; IFN, Interferon; IFNAR, type I interferon receptor; IL-1β, Interleukin 1β; IL-1ra, IL-1 receptor antagonist; iNOS, Inducible Nitric Oxidase Synthase; IPC, Ischemic Preconditioning; IRF, Interferon Regulatory Factor; KCl, Chloride potassium; LPS, lipopolysaccharide; MCAo, Middle Cerebral Artery occlusion; MCPIP1, Monocyte Chemotactic Protein–Induced Protein 1; MyD88, Myeloid differentiation primary response 88; NF-κB, Nuclear Factor-κB; NMDA, N-methyl-D-aspartate; NO, Nitric Oxide; ODN, oligodeoxynucleotide; Pam3CSL4, Pam3CysSerLys4; PGE_2_, Prostaglandin E2; PI3K, Phosphatidylinositol 3-Kinase; PI3KCA-γ, PI3K catalytic subunit gamma isoform; PMA, platelet–monocyte aggregates; PMN, polymorphonuclear; Poly-ICLC, polyinosinic polycytidylic acid; RANTES, Regulated on Activation, Normal T Cell Expressed and Secreted; S1P, Sphingosine-1-phosphate; Ship1, Src homology-2 domain-containing inositol 5- phosphatase 1; SNAP-23, Synaptosome-associated protein-23; SphK2, Sphingosine Kinase 2; TACE, Tumor Necrosis factor-alpha-Converting Enzyme; TIMP1, Tissue inhibitor of metalloproteinase 1; TLR, Toll-Like Receptor; TNFR, Tumor necrosis factor receptor; TNF-α, Tumor necrosis factor alpha; Tollip, toll interacting protein; TRIF, TIR-domain-containing adapter-inducing interferon-β*.

In this review we will focus on inflammatory pathways leading to cerebral IT. We will review the signaling cascades involved in immune activation in different PC modalities and highlight the molecular components involved. Lastly, this review will highlight the importance of epigenetic modifications and microRNAs in PC-induced reprogramming of the immune system.

## Evidence for a role of inflammatory pathways in different PC paradigms

A wide variety of preconditioning stimuli trigger the activation of inflammatory pathways that lead to brain IT (Table 2). The receptors, transducers and effector elements of these pathways are shared among different preconditioning paradigms. For example, TLRs are potent mediators of both ischemic (Pradillo et al., 2009; Wang et al., 2010) and endotoxic PC (acquired by systemic administration of LPS) (Tasaki et al., 1997; Ahmed et al., 2000; Vartanian et al., 2011). Likewise, the TNF pathway is involved in ischemic, hypoxic, hyperthermic and exercise-induced PC. Although inflammatory pathways are not independently sufficient for the induction of IT, there is evidence that several PC modalities are dependent upon the induction of such pathways.

### Ischemic, hypoxic, and hyperoxic PC

**Figure 1:**
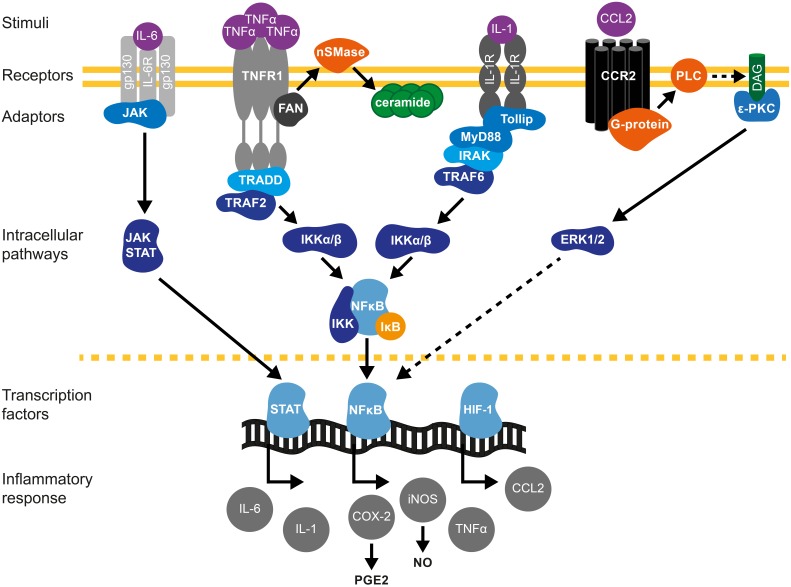
**Cytokine signaling pathways in brain preconditioning**. Following preconditioning (PC), the increase in tumor necrosis factor (TNF)-α, interleukin (IL)-1β and IL-6 and chemokine (C-C motif) ligand 2 (CCL2) leads to the activation of inflammatory cascades and induction of gene expression such as inducible nitric oxide synthase (iNOS) and cyclooxygenase (COX)-2, mediators of delayed PC. IL-6/IL-6R activates Janus kinase (JAK)-signal transducer and activator of transcription (STAT) pathway. Phosphorylation and nuclear translocation of STAT induces iNOS and COX-2 up-regulation (Dawn et al., 2004). TNF-α activates TNF receptor 1(TNFR1)/Tumor necrosis factor receptor type 1-associated DEATH domain protein (TRADD)/TNF-α receptor–associated factor 2 (TRAF2) pathway (Hallenbeck, 2002; Pradillo et al., 2005). Furthermore, TNF-α activation promotes ceramide synthesis that also mediates TNFα-preconditioning (Liu et al., 2000; Ginis et al., 2002). The neutral sphingomyelinase domain (NSD) of TNFR1 recruits factor associated with neutral sphingomyelinase activation (FAN). FAN activates neutral sphingomyelinase (nSMase) in the outer leaflet of the plasma membrane to produce ceramide (Hallenbeck, 2002). IL-1R activates toll interacting protein (tollip)/myeloid differentiation primary response 88 (MyD88)-IL-1R-associated kinase (IRAK)/TRAF6 signaling. TRAF mediates the activation of inhibitor of κB kinase (IKK), which triggers the phosphorylation-induced proteasomal degradation of the IκB, NF-κB activation and subsequent iNOS and COX-2 expression. CCL2/CCR2 activation promotes phospholipase-C (PLC)- diacylglycerol (DAG)-protein kinase C (PKC) pathway that leads to extracellular signal-regulated kinases (ERK1/2) activation and ultimately causes NF-κB activation (Rehni and Singh, 2012). In addition, HIF-1-induced expression of CCL-2 confers cerebral ischemic tolerance (Stowe et al., 2012). COX-2-derived prostaglandin E2 (PGE_2_) (Horiguchi et al., 2006) and iNOS-derived nitric oxide (NO) (Cho et al., 2005) are molecular mediators of PC.

Short non-damaging episodes of ischemia prior to a lethal ischemic episode leads to IT (Kirino, 2002). Cerebral IT can be acquired by local PC, mediated either by global (Kitagawa et al., 1990) or focal cerebral ischemia (Barone et al., 1998), or by remote ischemic PC (RIPC), typically achieved by limb ischemia (Tapuria et al., 2008). Although both TLR2 and TLR4 have been implicated in the post-ischemic inflammatory response (Cao et al., 2007; Abe et al., 2010; Brea et al., 2011; Shichita et al., 2012), only TLR4 has been shown to participate in ischemic preconditioning (IPC) (Konstantinov et al., 2004; Pradillo et al., 2009). TLR4 signaling activates transcription nuclear factor-κB (NF-κB) leading to the expression of TNF, inducible nitric oxide (iNOS) and cyclooxygenase-2 (COX-2), which are required to evoke a PC effect (see Table 2). Similarly, TNFR1, another central receptor of the innate immune system and activator of NF-κB, is essential for the induction of IPC (Konstantinov et al., 2004; Pradillo et al., 2005; Figure 1).

IT is not limited to the organ to which a stressor is applied. A PC stimulus applied to one organ also leads to IT in other organs, referred to remote PC (for reviews see Tapuria et al., 2008; Anrather and Hallenbeck, 2013; Hess et al., 2013). Remote ischemia can be achieved in rodents by transient occlusion of one or both femoral arteries, the mesenteric artery or the renal artery prior to cerebral ischemia. RIPC results in a decrease of the infarct volume following cerebral ischemia [for a comprehensive list in animal model see (Hess et al., 2013) and (Anrather and Hallenbeck, 2013)]. The effect of RIPC on circulating immune cells has been investigated in humans following forearm transient ischemia (Table 2). These studies showed that the expression of pro-inflammatory genes is down-regulated in circulating leukocytes (Konstantinov et al., 2004) and neutrophil function is altered (Shimizu et al., 2010).

Hypoxic-ischemic brain damage is mediated by TLRs (Stridh et al., 2011). Therefore, TLRs might also trigger inflammatory signaling in hypoxic PC (HPC), although no report to date has confirmed their involvement. To induce HPC in rodents, animals are placed in chambers and exposed to a hypoxic gas mixture, usually 8% oxygen and 92% nitrogen, for 3–4 h (Yin et al., 2007; Wacker et al., 2012). Hypoxia-inducible factor 1 (HIF-1) and NF-κB are activated in the brain after HPC (Yin et al., 2007; Wacker et al., 2012) leading to the expression of iNOS (Jung et al., 2000), TNF-α (Liu et al., 2000) and chemokine (C-C motif) ligand 2 (CCL2) (Stowe et al., 2012), which have been implicated in establishing IT after HPC (see below section Molecular Components of the Immune System Involved in PC).

The immune system is also involved in hyperoxic PC, which can be achieved by either normobaric hyperoxia or hyperbaric oxygen PC (HBO-PC). Delivering a gas mixture containing 90–95% oxygen induces normobaric hyperoxia. HBO-PC consists of the inspiration of an oxygen-containing gas mixture at higher than one atmospheric absolute pressure (ATA), usually 2–2.5 ATA. Bigdeli et al. showed that rats subjected to 16 h of normobaric hyperoxia had reduced infarct volume after Middle Cerebral Artery occlusion (MCAo) and that the TNF-α converting enzyme (TACE)/TNF-α/NF-κB pathway is associated with the protective effect (Bigdeli and Khoshbaten, 2008). Moreover, COX-2 has also been reported to be a mediator of HBO-PC (Cheng et al., 2011). Administration of the specific COX-2 inhibitor, NS-398, before HBO exposure reversed the protective effect of HBO-PC. Cheng et al. further identified COX-2-dependent upregulation of HIF-1α as a possible down-stream mediator of HBO-PC.

### Hypothermic and hyperthermic PC

Hypothermia induced during and/or after ischemia has a well-established protective effect in acute ischemic stroke (Wu and Grotta, 2013) and may be partly due the inhibition of the inflammatory response occurring during stroke (Ceulemans et al., 2010). Brief exposure to hyperthermia represents an additional method of PC (Table 2). In animal model of stroke, transient hypothermia prior an ischemic insult (Nishio et al., 2000; Yunoki et al., 2003) or during reperfusion (Kawai et al., 2000) reduces infarct volume. The latter study has reported a decrease number of polymorphonuclear leucocytes during reperfusion—shown by a reduced myeloperoxidase activity in rats subjected to hypothermia (Kawai et al., 2000). Hyperthermic PC reduces brain damage in newborn rats as well (Ota et al., 2000). The protective mechanism of hyperthermic PC has been linked to the induction of the heat shock protein 70 (HSP70) within glial and endothelial cells (Ota et al., 2000). In addition to its role as an intracellular chaperon, HSP70 acts as an endogenous ligand of TLR4. Extracellular HSP70 is released from damaged cells and serves as a damage-associated molecular pattern (DAMPs) molecule (Bianchi, 2007). It has been shown that HSP70 mediates endotoxin tolerance by signaling through the TLR4/CD14 pathway (Vabulas et al., 2002; Aneja et al., 2006), similar to the canonical TLR4 ligand LPS. Thus, induction of HSP70 following hyperthermia could be a factor in the establishment of IT.

### Anesthetic PC

Inhalation anesthetics are strong inducers of IT and can directly modulate immune functions in neutrophils, monocytes and lymphocytes (Clarkson, 2007; Wang et al., 2008). Rats pretreated with low dose isoflurane or halothane were protected after permanent MCAo (Kapinya et al., 2002). The protective effect was attributed to increased iNOS expression after anesthetic PC, and was reversed by treating the animals with the iNOS inhibitor aminoguanidine. Volatile anesthetics might directly alter the response of immune cells to inflammatory stimuli. Isoflurane PC of mouse macrophages induced the expression of heme oxygenase-1 (HO-1) and decreased NO and TNF-α release after subsequent LPS exposure (Li et al., 2009).

### Cortical spreading depression, brain stimulation, and seizures

CSD is an electrophysiological phenomenon characterized by a slowly propagating depolarization wave across the cortical surface that confers delayed IT (Yanamoto et al., 1998; Horiguchi et al., 2006; Shpargel et al., 2008). In animal models, CSD PC is elicited by either topical application or superfusion of the cortical surface with a 0.5–5 M KCl solution that induces IT lasting 1–15 days (Kawahara et al., 1995; Yanamoto et al., 1998; Horiguchi et al., 2006). The delayed tolerance induced by CSD is protein synthesis dependent and has been linked to the up-regulation of trophic factors and glial cell activation (Kawahara et al., 1999). On the other hand, spreading depression in organotypic hippocampal slice cultures increases the expression of several pro-inflammatory cytokines including TNF-α, IL-1β, IL-1α, and IL-6 (Kunkler et al., 2004). Indeed, *in vivo* experiments found an early induction of both TNF-α and IL-1β after CSD (Jander et al., 2001). Pre-treatment with the non-competitive N-methyl-D-aspartate (NMDA) receptor antagonist MK-801 completely abolished the upregulation of these cytokines, implicating NMDA receptors as a critical element driving their production. Additionally, Horiguchi et al. reported that CSD-induced neuroprotection against ischemic injury resulting from MCAo is dependent upon increased COX-2 expression (Horiguchi et al., 2005, 2006), supporting the involvement of the inflammatory response in CSD PC.

Electrical stimulation of the cerebellar fastigial nucleus, but not other cerebellar nuclei, can induce potent and long-lasting protection from focal or global cerebral ischemic injury (Reis et al., 1991, 1998; Golanov et al., 1998). At the same time, fastigial nucleus stimulation evokes a strong anti-inflammatory response and suppresses post-ischemic iNOS expression and brain inflammation after cortical IL-1β injection (Galea et al., 1998a,b). The effect is mediated, at least in part, by increasing the tolerance of mitochondria to calcium overload, and suppressing the release of mitochondrial pro-apoptototic factors induced by cerebral ischemia (Zhou et al., 2005). The molecular mechanisms of the protective effects on mitochondria involve upregulation of prohibitin, an integral protein of the inner mitochondrial membrane, which protects mitochondrial structure and function during cell stress (Zhou et al., 2012). Consistent with its role in PC, overexpression of prohibitin renders neurons more resistant to injury in a wide variety of models (Zhou et al., 2012).

Neuroprotection against cerebral damage induced by lethal ischemic/hypoxia or global ischemia can also be acquired through induction of mild epileptic activity elicited by kainic acid injections (Plamondon et al., 1999; Towfighi et al., 1999). Although the mechanisms are unknown, synthesis and release of TNF-α, IL-1β, IL-1α, and IL-6 by glia might contribute to this tolerance modality (Vezzani et al., 2002).

### Exercise PC

Physical exercise prevents stroke and forced exercise training affords neuroprotection against ischemic injury (Endres et al., 2003). Experimentally, exercise PC can be achieved by training rodents on a motor driven treadmill for 1–3 weeks (Wang et al., 2001; Ding et al., 2005; Curry et al., 2010). Among other mechanisms, such as protection against the blood-brain barrier (BBB) disruption, promotion of angiogenesis and inhibition of apoptosis, exercise PC involves activation of the immune system (Zhang et al., 2011). The skeletal muscle is an important source of several cytokines, often referred to as myokines, including IL-6, IL-8, IL-15, BDNF, LIF, and FGF21 (Pedersen, 2011). The release of these myokines into circulation might be responsible for the systemic effects of exercise, including its neuroprotective potential (Iadecola and Anrather, 2011b). Downstream mediators of exercise PC may include TNF-α (Ding et al., 2005) and extracellular signal-regulated kinases 1 and 2 (ERK1/2) (Guo et al., 2008; Curry et al., 2010). In one study, exercised rats submitted to MCAo showed reduced infarct volume when compared to non-exercised rats and this protection was associated with a gradual increase in the level of TNF-α in the brain over the 3 week course of treadmill exercise (Ding et al., 2005). Pre-ischemic blockage of TNF-α signaling with an anti-TNF-α antibody or inhibition of ERK1/2 activation abolished the protective effect (Guo et al., 2008). Exercise PC can also change the expression of innate immunity receptors. Treadmill exercise decreased cerebral TLR4 receptor expression in rats, although the importance of this finding as a mechanism for cerebral IT has not been investigated to date (Zwagerman et al., 2010).

## Molecular components of the immune system involved in PC

Inflammatory signaling is governed by a complex array of molecules that not only modulate the response of the target cell, but also change the local microenvironment and cellular tissue composition by recruiting cell types that are not normally present in that tissue. This inflammatory cascade is driven by cytokines, chemokines and their receptors and is regulated by immunomodulatory molecules. Several of these components have been implicated in PC. Evidence for direct involvement of some of these immune factors in cerebral IT is summarized in Table 3.

**Table 3:** **Evidence for the involvement of inflammatory mediators in PC**.

**Inflammatory mediators**	**Deletion/Inhibitor**	**PC type (dose)/time of application prior to brain ischemia**	**Index ischemia**	**Species**	**Outcome**	**References**
Chemokine receptors	CCL2 KO	HPC 2 days prior	tMCAo	Mouse	Reversed	Stowe et al., 2012
	CCL2 antibody	HPC 2 days prior	tMCAo	Mouse	Reversed	Stowe et al., 2012
Cytokines recpetors	TNF-α KO	LPS (0.2 mg/Kg) 3 days prior	tMCAo	Mouse	Reversed	Rosenzweig et al., 2007
	TNF-α KO	CpG ODN (1.6 mg/Kg) 3 days prior	tMCAo	Mouse	Reversed	Stevens et al., 2008
	TNFbp	LPS (0.2 mg/Kg) 2–4 days prior	pMCAo	SHR	Reversed	Tasaki et al., 1997
	IL-1ra	BCCo 3 days prior	BCCAo	Gerbil	Reversed	Ohtsuki et al., 1996
TLR receptors	TLR2 KO	Pam3CSK4 1 h prior	tMCAo	Mouse	Reversed	Lu et al., 2011
	TLR4 KO	BCCAo 2 days prior	pMCAo	Mouse	Partially reversed	Pradillo et al., 2009
	TLR7 KO	GDQ (40 μ g/mouse) 3 days prior	tMCAo	Mouse	Reversed	Leung et al., 2009
TLRs adaptors	TRIF KO	LPS (0.4 mg/Kg) 3 days prior	tMCAo	Mouse	Reversed	Vartanian et al., 2011
	IRF3 KO	LPS (0.5 mg/Kg) 3 days prior	tMCAo	Mouse	Reversed	Marsh et al., 2009b
	IRF3 KO	CpG ODN (1.6 mg/Kg) 3 days prior	tMCAo	Mouse	Reversed	Stevens et al., 2011
	IRF3 KO	tMCAo 3 days prior	tMCAo	Mouse	Partially reversed	Stevens et al., 2011
	IRF7 KO	LPS (1 mg/Kg) 3 days prior	tMCAo	Mouse	Reversed	Stevens et al., 2011
	IRF7 KO	CpG ODN (1.6 mg/Kg) 3 days prior	tMCAo	Mouse	Reversed	Stevens et al., 2011
	IRF7 KO	tMCAo 3 days prior	tMCAo	Mouse	Partially reversed	Stevens et al., 2011
iNOS	iNOS KO	tMCAo 1 day prior	tMCAo	Mouse	Reversed	Cho et al., 2005
	Amino- guanidine (iNOS)	tMCAO 1 day prior	tMCAo	Mouse	Reversed	Cho et al., 2005
	Amino- guanidine (iNOS)	LPS (0.5 mg/Kg) 1 day prior	tMCAo	Mouse	Reversed	Cho et al., 2005
	Amino- guanidine (iNOS)	Isoflurane 1 day prior	pMCAo	Rat	Reversed	Kapinya et al., 2002
COX-2	NS-398 (COX-2)	HBO 5 days prior	4VO	Rat	Reversed	Cheng et al., 2011
	Rofecoxib (COX-2)	tMCAO 8 h prior	pMCAo	Rat	Reversed	Park et al., 2008
NF-κB pathway	DTTC (NF-κB)	4VO 3 days prior	4VO	Rat	Reversed	Blondeau et al., 2001
	κB decoy DNA (NF-κB)	4VO 3 days prior	4VO	Rat	Reversed	Blondeau et al., 2001
	DTTC (NF-κB)	linolenic acid (500 nmol/Kg) 3 days prior	4VO	Rat	Reversed	Blondeau et al., 2001
	κB decoy DNA (NF-κB)	linolenic acid (500 nmol/Kg) 3 days prior	4VO	Rat	Reversed	Blondeau et al., 2001

*The deletion or inhibition of chemokines, cytokines, or TLR receptors, iNOS and COX-2 molecules and TLR or NF-κB signaling components described in the table, are able to abolish the preconditioning effect, unveiling the role of inflammatory pathways involved in IT*.

*4VO, 4 Vessel Occlusion; BCCAo, Bilateral Common Carotid Artery occlusion; COX-2, cyclooxygenase-2; CpG ODN, cytosine-guanine oligodeoxynucleotides; DTTC, diethyldithiocarbamate; GDQ, Gardiquimod; HBO-PC, Hyperbaric Oxygen Preconditioning; HPC, hypoxic preconditioning; IL-1ra, Interleukin 1 Receptor Antagonist; iNOS, inducible Nitric Oxide Synthase; IRF, Interferon (IFN)-regulatory factor; LPS, Lipopolysaccharide; pMCAo, permanent Middle Cerebral Artery occlusion; TLR, Toll-Like Receptor; SHR, Spontaneously Hypertensive Rats; tMCAo, transient Middle Cerebral Artery occlusion; TNFbp, Tumor Necrosis Factor binding protein; TNF-α, Tumor Necrosis Factor α; TRIF, TIR-domain-containing adapter-inducing interferon-β*.

### TLRs

The Toll receptor was first described in the context of Drosophila embryogenesis (Anderson et al., 1985). Subsequently, it was discovered that the Toll pathway is activated upon Drosophila fungal infection revealing a role of Toll receptors in the immune response (Lemaitre et al., 1996). Exposure of immune cells to certain pathogens activates these receptors resulting, in the activation of intracellular signaling pathways that lead to the expression of genes involved in the inflammatory response. In vertebrates, TLRs, named for their homology to the *toll* gene in Drosophila, are transmembrane proteins containing domains that are able to recognize viral or microbial components known as pathogen-associated molecular patterns (PAMPs) (Wang et al., 2011). Peptidoglycans (PGN), lipoproteins, lipoteichoic acids (LTA), lipopolysaccharides (LPS), as well as viral and bacterial nucleic acids, serve as PAMPs recognized by individual TLRs (Akira, 2009). To date 13 members of the TLR family have been identified in mammals. Among the most extensively studied TLRs are TLR2 which binds to LTA component of Gram-positive bacteria and TLR4, which recognizes LPS on the cell wall of Gram-negative bacteria (Verstak et al., 2007). Upon ligand binding, TLRs further signal by recruiting intracellular Toll/IL-1R (TIR)-homology domain containing adaptor proteins, which selectively activate signaling cascades that lead to immune responses. Several TLR adapter molecules that are associated with functionally different signaling cascades, such as myeloid differentiation factor-88 (MyD88) and TIR domain containing adaptor protein inducing interferon β (TRIF), are known to date. With the exception of TLR3, TLRs signal through MyD88-dependent pathways, while TLR4 can activate both MyD88-dependent and—independent pathways (Wang et al., 2011; Mallard, 2012).

MyD88 mobilizes members of the IL-1R-associated kinase family (IRAK) leading to nuclear translocation of the transcription factor NF-κB. NF-κB is a major regulator of the inflammatory response (Li and Verma, 2002) and is responsible for the induction of various inflammatory genes including cytokines, chemokines, adhesion molecules, proinflammatory enzymes, and growth factors (Pahl, 1999). NF-κB is commonly found as a heterodimer consisting of p50 (NFKB1) and p65 (RelA) subunits. In resting cells, the inactive form of NF-κB is located in the cytoplasm by association with inhibitor proteins, IκBα and IκBβ (Baeuerle and Henkel, 1994). Upon stimulation, IκB proteins are phosphorylated and proteolytically degraded, and NF-κB dimers are able to translocate to the nucleus and bind to the promoter region of target genes initiating their transcription (Baldwin, 1996). NF-κB is activated in many cell types including cells of the central nervous system (Kaltschmidt et al., 1994). In the rodent brain, NF-κB can be activated by numerous factors known to be induced after ischemia-reperfusion, such as glutamate, increased intracellular Ca^2+^, reactive oxygen species (ROS) and inflammatory cytokines (Harari and Liao, 2010). Furthermore, target genes of NF-κB have been implicated in the pathogenesis of cerebral ischemia, such as IL-1, IL-6, TNF-α, ICAM-1, MMP9, COX-2, and iNOS (Baeuerle and Henkel, 1994; Allan et al., 2005; Kaltschmidt et al., 2005; Harari and Liao, 2010). Thus, it has been postulated that stimulation of signaling pathways that lead to NF-κB activation are associated with detrimental outcome in cerebral ischemia (Stephenson et al., 2000). Supporting this notion, inhibition of NF-κB using pharmacologic agents results in reduced infarct size after stroke in rodents (Clemens et al., 1998; Nurmi et al., 2004). It has also been shown that mice lacking the p50 NF-κB subunit have reduced ischemic damage (Schwaninger et al., 1999; Nurmi, 2004). However, subsequent studies have also revealed a deleterious effect of NF-κB inhibition in neonatal hypoxia (Nijboer et al., 2008) and an increase of neuronal death after transient ischemia in p50 deficient mice (Duckworth et al., 2006) raising the possibility for a dual role of NF-κB in stroke.

**Figure 2:**
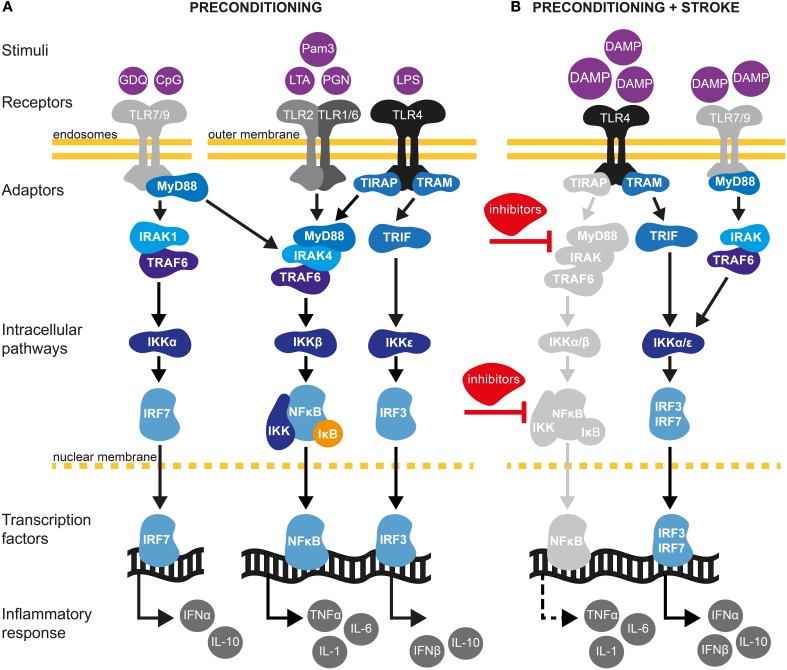
**TLR signaling pathways in cerebral IT. (A)** Preconditioning through TLRs may be afford by exposure with low dose of pathogen-associated molecular pattern (PAMPs) molecules (Mallard, 2012). TLRs are localized either at the outer cell surface (TLR1, TLR2, TLR4, and TLR6) or within endosomes (TLR7 and TLR9) (Marsh et al., 2009a). TLR2 dimerizes with TLR1 or TLR6 and is activated upon binding of PAMPs such as the synthetic lipopeptide Pam3CysSerLys4 (Pam3CSK4), the non-peptide ligand lipoteichoic acid (LTA) or peptidoglycans (PGN). TLR4 predominantly recognizes lipopolysaccharides (LPS) from Gram-negative bacteria. TLR7 and TRL9 can be activated by the synthetic imidazoquinoline Gardiquimod (GDQ) or small DNA such as non-methylated cytosine-guanosine (CpG), respectively (Wang et al., 2011). Preconditioning of TLRs will favor the activation of the myeloid differentiation factor-88 (MyD88)-dependent pathway. Upon binding of ligands, MyD88 is activated either directly (TLR7/9) or via the toll-interleukin 1 receptor (TIR) domain containing adaptor protein TIRAP (TLR2/4). The latter further mobilizes members of the IL-1R-associated kinase family (IRAK) leading to the subsequent binding/activation of TNF receptor associated factor-6 (TRAF6), the inhibitor of κB kinase (IKK) complex (composed of IKKα and IKKβ) and IκB. Once IκB is phosphorylated and degraded by the proteasome, nuclear factor-κB (NF-κB) translocates to the nucleus leading to low level of pro-inflammatory cytokine synthesis, such as TNF-α. TLR PC also induces the production of several negative inhibitors of TLR signaling, targeting mainly MyD88 and NF-κB pathways. Induction of proinflammatory cytokines and activation of such inhibitors are required to induce a state of ischemic tolerance (Marsh et al., 2009a; Vartanian and Stenzel-Poore, 2010; Wang et al., 2011). In addition to activating the MyD88-dependent pathway, TLR4 signals through the MyD88-independent pathway to activate IRF3 by sequential recruiting of the adaptor molecule TIR inducing interferon β (TRIF) and IKKε resulting in expression of anti-inflammatory type I interferon (IFN) genes, including IFNβ (Marsh et al., 2009b). Type I IFNγ genes are also induced by TLR7/9 activation through the TRAF6/IKKα pathway (Hoshino et al., 2006). **(B)** Damage-associated molecular pattern (DAMPs) molecules are endogenous ligands of TLRs produced in response to ischemic injury (Vabulas et al., 2002). Following preconditioning, activation of TLR4/7/9 by DAMPs will fail to activate MyD88 and the subsequent signaling molecules, such as IRAK and NF-κB. Indeed, several inhibitors produced during the first exposition of TLRs to exogenous stimuli will block the signaling of TLRs to NF-κB. Whether pro-inflammatory cytokines are suppressed or up-regulated is controversial and might be model specific (Marsh et al., 2009a; Vartanian and Stenzel-Poore, 2010). On one hand, TLR preconditioning induces a down-regulation of NF-κB. On the other hand, it has been shown that neuroprotection following cerebral ischemia is achieved by an up-regulation of the TLR-IRF axis generating an increase of TGF-β, IL-10, IFNα, and IFNβ anti-inflammatory cytokines (Stevens et al., 2011; Leung et al., 2012). Thus, TLR preconditioning induces a reorganization of the TLR signaling pathways after cerebral ischemia toward TRIF/IRF signaling that may confer protection of the brain against ischemic damage.

TLR4 stimulation also leads to the activation of MyD88-independent pathways. In this case, binding of the adaptor molecule TRIF activates the transcription factors interferon regulatory factor 3 (IRF3) and 7 (IRF7), resulting in expression of IFNα and IFNβ type I IFN genes (Marsh et al., 2009a; Wang et al., 2011; Mallard, 2012) (Figure 2). Type I IFNs are released in the extracellular space and signal in an auto- or paracrine manner through a single heterodimeric receptor, IFNAR, to activate the JAK/STAT pathway, leading to the expression of several chemokines such as CCL2, CCL7, and CXCL10, while inhibiting CXCL1 and CXCL2 expression. Depending on the concentration of IFN and type of target cell, the resulting immune response can be pro- or anti-inflammatory (Trinchieri, 2010).

Aside from binding to PAMPs, TLRs also recognize endogenous molecules known as DAMPs, which are released during ischemic cellular injury (Chen and Nuñez, 2010). Prototypical DAMPs released from injured cells include high-mobility group box 1 (HMGb1) protein that activates TLR2, 4, and 9; heat shock proteins (HSPs; TLR2, 4); RNA (TLR3); mitochondrial DNA (TLR9); hyaluronic acid (TLR2, TLR4) and peroxiredoxins (TLR2, TLR4) (Chen and Nuñez, 2010; Patel et al., 2012; Shichita et al., 2012). HMGb1 and peroxiredoxins have been reported to increase cerebral ischemic injury by augmenting the post-ischemic inflammatory response (Kim, 2006; Yang et al., 2011; Shichita et al., 2012). While the role of DAMPs in cerebral IT has not been addressed, it has been shown that systemic administration of HMGb1 protects against myocardial ischemia–reperfusion injury and liver ischemia-reperfusion injury, a process that involves TLR4 (Izuishi et al., 2006; Hu et al., 2010). Additionally, HSP70 mediates endotoxin tolerance by signaling through TLR4 receptors (Aneja et al., 2006). Therefore, these findings support DAMPs to participate in the PC effect through TLRs activation. In contrast to the detrimental effect of TLR activation in response to ischemia (Wang et al., 2011), stimulation of some TLRs prior to ischemia provides robust neuroprotection (Marsh et al., 2009a; Wang et al., 2011). The ability of TLR ligands to induce cerebral IT was first demonstrated after systemic administration of low-dose LPS, the major TLR4 ligand, causing spontaneously hypertensive rats to become tolerant to subsequent ischemic brain damage induced by MCAo (Tasaki et al., 1997). Subsequently, LPS-induced tolerance to brain ischemia has been demonstrated in several experimental stroke models in mice, gerbils and pigs (Rosenzweig et al., 2004; Hickey et al., 2007; Yu et al., 2010). A dose below 1 mg/Kg of LPS administered 1–3 days prior to cerebral ischemia is generally used in rodents to induce IT. Furthermore, TLR4-mutant mice were refractory to IPC, indicating that TLR4 plays an universal role in establishing IT (Pradillo et al., 2009). Comparable to TLR4, pre-treatment with the TLR2 lipopetide ligand Pam3CysSerLys4 (Pam3CSK4) resulted in significantly decreased cerebral infarct volume after focal cerebral ischemia/reperfusion in mice (Hua et al., 2008). Preservation of the BBB has been suggested to play a role in this PC mechanism. Similarly, stimulation of TLR7 by administration of its agonist gardiquimod, an imidazoquinoline compound, provides neuroprotection against ischemic injury and was associated with the up-regulation of the IFN pathway and IFNα production (Leung et al., 2012). Interestingly and in contrast to TLR4 and TLR9 induced PC, TLR7-mediated PC was abolished in IFNAR mutant mice. Finally, activation of TLR9 by unmethylated CpG oligodeoxynucleotides induced IT after transient MCAo in mice (Stevens et al., 2008). Similar to LPS-PC, the protective effect after TLR9 stimulation lasted for up to a week and was dependent on the expression of TNF-α (Figure 2A) (Stevens et al., 2008; Marsh et al., 2009a). Accordingly, CpG oligodeoxynucleotides administration to TNF-α null mice failed to induce IT.

IT induced by TLR activation involves the reprogramming of inflammatory pathways. This is achieved by at least two different mechanisms. Concomitantly with induction of pro-inflammatory mediators, activation of the MyD88-dependent pathway will result in the upregulation of negative regulators, which prevent the interaction of several adaptors along the TLR4-MyD88 pathway (Figure 2A) (Sly et al., 2004). At the same time, activating the MyD88-independent pathway evokes a type I IFN response. Indeed, recent studies have found a neuroprotective role of the MyD88-independent pathway in TLR-induced IT after stroke (Marsh et al., 2009a). It has been postulated that pretreatment with LPS induces a switch in the transcriptional response to subsequent TLR4 stimulation by increasing the expression of the IRF3-induced cytokine IFNβ (Veldhuis et al., 2003) (Figure 2B). Consistent with this hypothesis, gene expression analysis following LPS-PC revealed upregulation of inhibitors of the Myd88-dependent and NF-κB pathways (Vartanian et al., 2011). Interestingly, the expression of pro-inflammatory genes related to the NF-κB signaling pathway was similar in both mice undergoing LPS-PC and in control mice following cerebral ischemia (Figure 2B). However, the study showed that LPS-PC increased IRF3 activity following ischemia and evoked the upregulation of anti-inflammatory genes including TGF-β and IL-10 (Vartanian et al., 2011). The importance of the MyD88-independent pathway in establishing IT is further supported by the fact that LPS-PC was unable to induce IT in TRIF- but not in Myd88-deficient mice (Vartanian et al., 2011).

### Cytokines

Cytokines are secreted proteins with growth, differentiation, and activation functions that shape the nature of the immune response. Among the more than 70 candidate cytokines, TNF-α, IL-1, IL-6, and IL-17 play major roles in initiating and amplifying the post-ischemic inflammatory response, while IL-10 and TGF-β are the main anti-inflammatory factors (Iadecola and Anrather, 2011a). Since immune cells, including monocytes/macrophages, in the periphery and microglia in the brain express TLR receptors (Downes and Crack, 2010), they are capable of responding to systemic TLR agonists used as a preconditioning stimulus and modify cytokine expression or secretion (Rosenzweig et al., 2004; Ransohoff and Brown, 2012). Accordingly, several cytokines secreted by cells of the innate immune system have been implicated in PC. For example, TNF-α has been identified as an essential mediator of LPS-PC. It has been shown that LPS-preconditioned TNF-α null mice were not protected from ischemic brain injury (Rosenzweig et al., 2007). This study confirmed previous findings demonstrating that administration of a specific TNF antagonist reversed the protective effect of LPS PC in a model of permanent focal ischemia in mice (Tasaki et al., 1997). Similarly, upregulation of IL-1α and IL-1β after bilateral common carotid artery occlusion (BCCAo) triggered tolerance to global ischemia (Ohtsuki et al., 1996; Shin et al., 2009). Ohtsuki et al. also showed that systemic delivery of either IL-1α or IL-1β was sufficient to induce IT to a subsequent episode of lethal global ischemia and that intraperitoneal injection of recombinant human IL-1 receptor antagonist prior to IPC abolished the neuroprotective effect.

In addition to systemic administration of cytokines, neuroprotection has also been reported with direct delivery of cytokines to the brain before ischemia. Previous intracerebrovascular injection of IL-6 in rats subjected to permanent ischemia protects from cell death (Loddick et al., 1998). Likewise, intracisternal administration of TNF-α in mice before distal MCAo is neuroprotective (Nawashiro et al., 1997). However, in the same study, both intravenous and intraperitoneal routes of TNF-α administration failed to exert neuroprotection against cerebral ischemia. While differences in modality of administration and stroke models may account for the observed disparities in outcome, it remains unclear how systemic cytokines exert their effect within the brain. TNF-α, IL-1β, and IL-6 are transported unidirectionally across the BBB (Banks, 2005). Thus, systemic induction of cytokines can lead to an increase of them in the brain. For instance, IL-1 expression in the brain has been documented after an increase of circulating IL-1 induced by LPS PC (Gabellec et al., 1995). On the other hand, inducers of PC, such as LPS and cytokines, can act through interaction with receptors in circumventricular organs that lack a BBB, triggering upregulation of cytokine levels in the brain. Accordingly, it has been shown that perivascular cells and neurons in the circumventricular organs produce TNF-α in response to systemically administered LPS (Breder et al., 1994).

### Chemokines

Chemokines are small molecules involved in the recruitment of immune cells to sites of inflammation or injury (Charo and Ransohoff, 2006). In the central nervous system, chemokines are highly upregulated in neuropathologic conditions. Neurons, astrocytes, microglia, endothelial cells as well as circulating leukocytes are potential sources of chemokines. Several chemokines have been linked to the pathogenesis of ischemic brain injury (Jaerve and Müller, 2012). Upon release, they exacerbate tissue injury by increasing leukocyte and monocytes/macrophages infiltration, potentiating neuronal injury. However, chemokines also possess neuroprotective and neurotrophic functions (Semple et al., 2009). The prevalence of beneficial or detrimental effects may depend on various factors, including the type of the chemokine, its concentration, the time course of production relative to the time of injury and the target cell type (Semple et al., 2009). The contribution of chemokines to PC has been investigated in several models of IT. For instance, HPC or BCCAo prior to transient or global MCAo resulted in reduced infarct size attributed to CCL2 (MCP-1), a leukocyte-derived pro-inflammatory chemokine (Rehni and Singh, 2012; Stowe et al., 2012). Following HPC, expression of CCL2 increased in both neurons and cerebral endothelial cells prior to the induction of the ischemic injury. Moreover, the induction of IT was blocked by CCL2 neutralizing antibodies and was not observed in CCL2-knockout mice (Stowe et al., 2012). Although the protection conferred by HPC is associated with the number of CCR2^+^ monocytes in blood and leaving the circulation, their direct protective role in brain IT needs to be further characterized. Rehni et al. have shown that IT induced by BCCAo PC was lost after treatment with a selective antagonist of CCR2 (Rehni and Singh, 2012), corroborating the role of CCL2 chemokines and its receptor in IT.

### Cannabinoids

The endocannabinoid system consists of the cannabinoid type 1 (CB1) and type 2 (CB2) receptors and their ligands (Howlett et al., 2002). The expression of these two receptor subtypes varies among tissues. CB1 receptors are abundant in the CNS and are also present in several peripheral tissues, albeit to a lesser extent (Matsuda et al., 1990; Galiègue et al., 1995). Although also present in the brain (Gong et al., 2006), CB2 is mostly expressed in immune cells (Galiègue et al., 1995). Cannabinoid receptors may be activated upon binding of their endogenous ligands arachidonoyl ethanolamide or anandamide (AEA), 2-arachidonoylglycerol (2-AG), and 2-arachidonyl glyceryl ether (noladin ether), or synthetic analogs which are derivatives of herbal cannabinoids, such as the terpenoid Δ^9^-tetrahydrocannabinol (THC), the active compound of the cannabis plant (Di Marzo et al., 1998). Both CB1 and CB2 receptors are members of the seven-transmembrane G-protein coupled receptor superfamily. They may initiate downstream signaling pathways that activate potassium channels, phosphatidylinositol-3-kinase and mitogen-activated protein kinases (Di Marzo et al., 1998). The CNS endocannabinoid system has a variety of physiological roles, which are mainly mediated by CB1 receptors. These include, but are not limited to: psychotropic effect, pain inhibition, memory function and increased appetite (Ameri, 1999; Pacher and Haskó, 2008). Modulation of immune responses and the release of inflammatory mediators have been primarily attributed to CB2 receptors (Pacher et al., 2006; Ullrich et al., 2007; Elliott et al., 2011).

The endocannabinoid system has been implicated in ischemic injury (Pacher and Haskó, 2008). Several studies have shown a neuroprotective effect of CB activation in brain ischemia (Nagayama et al., 1999; Panikashvili et al., 2001) by decreasing intracellular Ca^2+^ (Zhuang et al., 2005), modulating brain temperature (Leker et al., 2003), inhibiting pro-inflammatory signaling cascades (Panikashvili et al., 2005) and by preventing endothelial cell activation and leukocyte adhesion (Zhang et al., 2007, 2009). There is emerging evidence that cannabinoids also play a role in PC. Induction of IT by electroacupuncture PC improves neuronal survival in a rat model of focal ischemia (Wang et al., 2005, 2009; Ma et al., 2011). While early PC in this model was dependent on the activation of CB1 receptors as demonstrated by the reversal of the protective effect after administration of the CB1 receptor antagonist AM251 or CB1 siRNA (Wang et al., 2009), delayed PC was dependent upon the activation of CB2 receptors (Ma et al., 2011). It was shown that IT was partially reversed when animals were treated with the specific CB2 antagonist AM630. Inhibition of the CB1 receptor, however, did not block the induction of delayed IT. Although the cellular components of this response have not been investigated, the nearly exclusively expression of CB2 receptors on immune cells makes it possible that modulation of the immune response accounts for the observed neuroprotection.

### iNOS

Nitric oxide (NO) is produced by nitric oxidase synthase (NOS) through oxidation of the guanidino nitrogen of L-arginine. Endothelial (eNOS), neuronal (nNOS) and inducible or inflammatory (iNOS) isoforms are found in the brain, and play important roles in IT (for recent review see (Iadecola et al., 2011). iNOS is specifically expressed under pathological conditions, typically those associated with inflammation (Nathan, 1997). Data from our laboratory have shown that iNOS mediates IT following either IPC or LPS-PC in a mouse model of transient MCAo (Cho et al., 2005). PC-mediated neuroprotection was abolished when the selective inhibitor, aminoguanidine, was delivered prior to PC, or when iNOS null mice were used. iNOS expression and accumulation of peroxynitrite in cerebral blood vessels was observed 24 h after IPC (Cho et al., 2005), whereas LPS-PC was associated with accumulation of peroxynitrite in neurons and vessels (Kunz et al., 2007). The role of iNOS in PC has also been reported with anesthetic preconditioning (Kapinya et al., 2002). Prior treatment with isoflurane or halothane, which reduces infarct volume after permanent MCAo, induced iNOS expression in the cortex 18–24 h after PC. Analogous to the studies mentioned above, anesthetic PC was blocked when animals were treated with the iNOS inhibitor aminoguanidine.

### COX-2

Similar to iNOS, brain cycloxygenase-2 (COX-2) is markedly upregulated during post-ischemic inflammation and its reaction products contribute to the evolution of ischemic damage (Iadecola et al., 2001). Likewise, the role of COX-2 has been investigated in several PC paradigms. Increased brain levels of COX-2 following PC by CSD, was associated with the development of cerebral IT in a rat model of MCAo (Horiguchi et al., 2006). IPC by transient MCAo induced COX-2 and HO-1 expression, and significantly reduced infarct volume after index MCAo. Administration of the COX-2-selective inhibitor rofecoxib abolished the neuroprotective effect of IPC, indicating a key role of COX-2 in establishing IT (Park et al., 2008). More recently, the role of COX-2 in PC induced by HBO has also been reported (Cheng et al., 2011). COX-2 induction is associated with protection against subsequent global cerebral occlusion in rats and IT was abolished by treatment with COX-2 selective inhibitor NS-398. Contrary to IPC, no role of COX-2 in LPS PC was found in a excitotoxic model of brain injury induced by NMDA (Kawano et al., 2007).

### ROS

ROS play a key role in the pathogenesis of the ischemic cascade. ROS are mainly generated after cerebral ischemia by the injured tissue during the acute phase (minutes-hours) and by infiltrating leukocytes during the sub-acute ischemic phase (Chan, 2001; Kahles et al., 2007). For example, neutrophils that infiltrate the ischemic brain produce excessive superoxide via NADPH oxidase, contributing to the exacerbation of ischemic injury (Kunz et al., 2006; Chen et al., 2009; Pun et al., 2009). In preconditioning, the role of ROS via inflammatory mechanisms has also been reported. Kunz et al. showed improvement of cerebrovascular function after transient MCAo in LPS preconditioned mice, an effect associated with peroxynitrite formation (Kunz et al., 2007). Interestingly, peroxynitrite was specifically formed after PC from the reaction of iNOS-derived NO and Nox2 (NADPH oxidase)-derived superoxide, and was not observed in iNOS or Nox2-null mice. Moreover, peroxynitrite was also found to be beneficial in the tolerance induced by LPS-PC to brain injury resulting from cortical injection with NMDA (Kawano et al., 2007).

## Epigenetic mechanisms

In delayed PC, the time window during which IT is observed can last for days or even weeks. This contrasts with the limited duration of inflammatory signaling where responses are often short lived, and several negative feedback loops are in place to restrict the duration of the inflammatory response. This is achieved by the expression of a series of cellular inhibitors that are concomitantly produced along with pro-inflammatory molecules, leading to a timely termination of gene expression after the stimulus subsides. These inhibitors can directly target transcription factors, as is the case with NF-κB, which is sequestered in the cytoplasm by newly synthetized IκB proteins—or, by inactivating key proteins within the pro-inflammatory signaling cascade. For example, A20, an ubiquitin editing enzyme that is highly inducible by pro-inflammatory stimuli, targets several proteins in the TNFR signaling cascades by removing K63-linked ubiquitin chains from TRAF2, TRAF6 and NEMO. It results in suppression of NF-κB signaling, and by replacing K63- with K48-linked ubiquitin chains in RIP1, thus targeting it for proteasomal degradation (Verstrepen et al., 2010). Similarly, suppressor of cytokine signaling (SOCS), a family of 40 related proteins, and PIAS (protein inhibitor of activated STAT) target primarily the JAK (Janus kinase)/STAT (signal transducer and activator of transcription) pathway, which is the main cellular transducer of cytokine signals (Yoshimura et al., 2007). Accordingly, short-acting inflammatory signals, as applied during PC protocols, are transient, and not suitable to alter the cellular state for an extended period of time. This view is supported by experimental data in ischemic and LPS preconditioned animals, that show transient global alterations of gene expression profiles after PC, which have largely dissipated by the time of index ischemia (Stenzel-Poore et al., 2003; Marsh et al., 2009b). This raises the question of how the “memory” of the PC event is preserved if the attendant mRNA response has already subsided. An essential feature of such a mechanism, is its persistence beyond the initial stimulation and its decay over time, thus, equipping the cell or organ with a relatively short lived memory of a previous exposure to a potentially lethal stressor. One possibility is that the protein products of the subsided transcriptional activity are still present at the time of induction of ischemia, which could steer the response from injury toward protection. Another possibility is that the transcriptional response to PC induces longer lasting epigenetic changes that shape the genetic response when injurious ischemia occurs. Epigenetic mechanisms include all processes that regulate gene expression without alteration of the underlying DNA sequence, and include, for example, histone modifications, DNA methylation, and non-coding RNAs.

### Histone and DNA modifications in preconditioning of the immune system and the brain

Histone modifications by methylation, acetylation, phosphorylation, ubiquitination, and sumoylation are key events in regulating chromatin structure and gene expression. The mechanism by which these modifications alter gene expression is not entirely understood, but may involve tethering of chromatin remodeling machinery, transcriptional repressors and activators. While acetylation of N-terminal histone tails are a hallmark of actively transcribed genes, it is less likely that histone acetylation plays a major role in imprinting a lasting memory from a previous exposure. Supporting this view is the fact that histone deacetylase (HDAC) is recruited early to the promoter of induced genes, in most cases during the gene activation process itself, resulting in a high turnover rate of acetylation marks in the order of minutes and fast disposal of this modification once transcription of the particular gene subsides (Hazzalin and Mahadevan, 2005). On the other hand, DNA methylation is a very thermostable epigenetic mark and, with the possible exception of 5-hydroxymethylcytosine, refractory to environmental stimuli. Therefore, DNA methylation is unsuited to act as an on-off switch for epigenetic memory (Qureshi and Mehler, 2010). Histone H3 tri-methylation at lysine 4 (H3K4me3), in contrast, has been shown to mark active or poised enhancers for an extended period of time. In a model of Candida albicans infected macrophages, persistent H3K4me3 modifications in the enhancer regions of inflammatory genes was associated with a more robust inflammatory response and reduced re-infection rate upon re-exposure (Quintin et al., 2012). Notably, the K4 trimethylation was observed for up to a week after deposition. The importance of H3K4me3 in changing transcriptional responses has been shown in LPS-preconditioned macrophages (Foster et al., 2007). Macrophages pre-exposed to LPS responded to a second LPS exposure with a transcriptional program distinct from that of naïve macrophages exposed to LPS. Genes could be divided into “tolerizeable” genes (T), which were suppressed in tolerant macrophages, and “non-tolerizeable” genes (NT), expressed at higher levels in tolerant than non-tolerant macrophages. It was found that promoter regions of T-class genes were refractory to histone H4 acetylation and H3K4 trimethylation, two modifications positively correlated with transcriptional activity, upon the second LPS challenge (Foster et al., 2007). Depending on the chromatin status in the resting state, genes can be divided in primary and secondary response genes (Ramirez-Carrozzi et al., 2006). Primary genes show a chromatin structure that is suited for immediate transcription and their activation is independent of new protein synthesis. Secondary genes show histone modifications characteristic of inactivated genes and the removal of this block and gene induction is protein synthesis dependent. Interestingly, this study found that LPS-PC changed many genes from secondary to primary response genes upon LPS re-stimulation, further implicating that epigenetic memory has been established in this model.

Several histone modifiers have been implicated in the PC response of the brain, including sirtuin family HDACs (Morris et al., 2011) and polycomb group proteins (PcG), a multimeric protein complex that mediates gene silencing and PC (Stapels et al., 2010). In addition to their role in neuronal physiology, both protein families have been implicated in the regulation of immune function (Swigut and Wysocka, 2007; Gallí et al., 2011). Whether they also regulate the immune signaling during PC, however, has not been directly addressed thus far.

### microRNAs and long non-coding rNAS

microRNAs (miRNAs) are small non-coding RNAs (18- to 24- nucleotides) that repress translation of mRNAs. Mature miRNAs are excised from a precursor RNA by the activity of Dicer and are guided to the 3′-untranslated region (3′-UTR) of mRNAs targets by the RNA-induced silencing complex (RISC). This leads to the down-regulation of gene expression via degradation or translational inhibition (Bartel, 2009). miRNAs have an essential quality that makes them compatible with the maintenance of preconditioning memory, namely their stability (Rüegger and Großhans, 2012). In addition, they are inducible by environmental stimuli, and are regulated at the levels of transcription, biogenesis, stability and decay. Supportive for a role of some miRNAs in establishing epigenetic memory, is the fact that they are extremely long lived, more than 12 days in the case of miR-208 in the heart (van Rooij et al., 2007). Furthermore, ablation of many miRNAs is well tolerated in the mouse, with these mutant animals only developing substantially altered phenotypes after being subjected to some kind of stress. These observations suggest that the persistence of inducible miRNAs due to their long half-lives after initial stimulation, may enable the maintenance of gene-expression programs that enhance the resistance of cells to repeated stress-exposure. When such “memory” miRNAs are absent (such as in animals depleted of the genes encoding the miRNAs), aberrant phenotypes become apparent (Leung and Sharp, 2010). The findings that miRNA can be actively secreted and transferred from cell-to-cell via microvesicles makes it possible that miRNA can work at a distance by transferring epigenetic signatures from a stimulus-exposed cell to a cell that has not encountered the particular environmental stressor (Valadi et al., 2007).

It is estimated that 500–1000 different miRNAs are expressed in mammalian cells and several miRNAs have been implicated in the regulation of the immune response and inflammation (O'Connell et al., 2012). Certain miRNAs, for instance, miR-9, -21, -146a, -147, -203, are induced in response to TLR activation, and serve as negative feedback regulators of the MyD88-dependent TLR signaling pathway and NF-κB activity (O'Neill et al., 2011). Conversely, miR-155 enhances the inflammatory response by down-regulating negative regulators Src Homology-2 domain-containing inositol-5′-phosphatase 1 (SHIP1) and SOCS1 leading to prolonged activation of AKT and IFN signaling pathways (O'Connell et al., 2009).

Cerebral ischemia-reperfusion injury alters the expression of several miRNAs (Dharap et al., 2009; Wang et al., 2013). Interestingly, IPC and HPC have been shown to rapidly alter miRNA levels in the brain (Dharap and Vemuganti, 2010; Lee et al., 2010; Liu et al., 2012). Although it is unknown if these alterations directly affect immune pathways participating in PC, the finding that expression of miRNAs involved in inflammatory signaling is altered by IPC in mice, is suggestive for such a role. For example, miR-17-5p, an inhibitor of monocyte maturation, and the TLR4-targeting miRNA let-7e were down-regulated in the cerebral cortex of mice after IPC (Lusardi et al., 2010). Furthermore, the peroxisome proliferator-activated receptor gamma (PPARγ)-targeting miR-27b and miR-146a, which regulates Traf6, IRAK1, and IRAK2 expression, were up-regulated after PC by transient MCAo in rats (Dharap and Vemuganti, 2010). The same study showed down-regulation of miR-145, which targets the essential TLR4-associated signaling molecule MyD88-adapter-like (MAL). Tellingly, monocyte chemotactic protein-induced protein 1 (MCPIP1), a multifunctional protein with RNAse activity that antagonizes Dicer, and thereby inhibits miRNA biogenesis (Suzuki et al., 2011), has been shown to abrogate the effects of LPS-PC in a mouse model of transient cerebral ischemia (Liang et al., 2011). Taken together, although PC-induced regulation of immune components by miRNA has not been conclusively shown to contribute to cerebral IT, the correlative data gathered so far are supportive for a role of miRNAs in regulation of immune pathways during PC.

Long non-coding RNAs range from 200 bp to several kb, and are expressed in a cell type specific manner (Mercer et al., 2009). Although most of these molecules have not been functionally characterized, they may have diverse roles ranging from regulation of chromosome inactivation to control of gene expression (Mercer et al., 2009). Because several long non-coding RNAs have been shown to be inducible and are involved in regulating immune cell activity and inflammatory signaling, they constitute another potential player by which epigenetic processes can establish a cellular memory. Their role in PC has yet to be investigated.

## Conclusions

**Figure 3:**
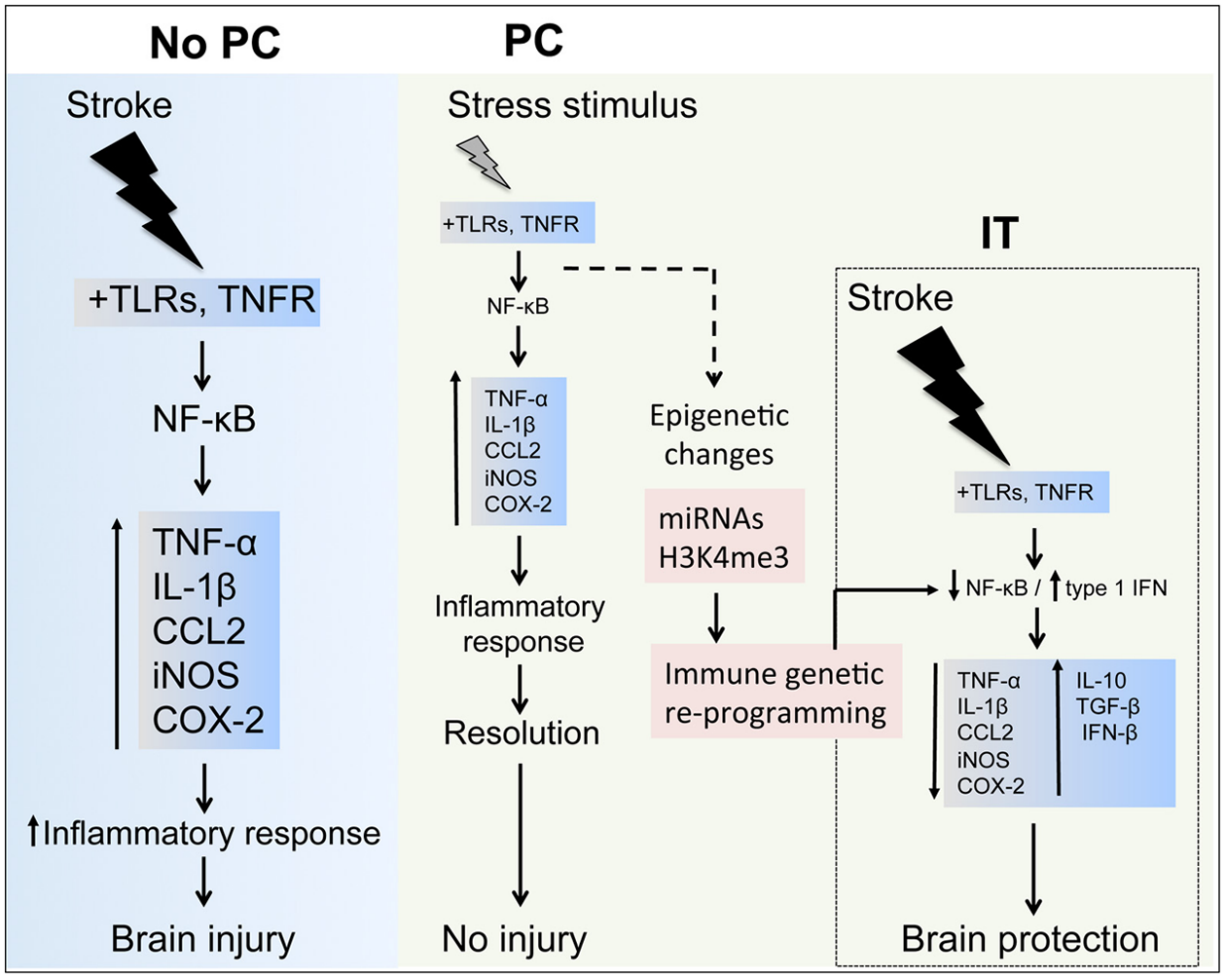
**Inflammatory components in stroke and preconditioning**. Stroke induces a major inflammatory response through toll-like receptors (TLRs) and tumor necrosis factor receptor (TNFR) signaling, which activate nuclear factor-κB (NF-κB) resulting in upregulation of inflammatory molecules, such as TNF-α, interleukin (IL)-1β, CCL2, inducible nitric oxidase synthase (iNOS) and cyclooxygenase (COX)-2, contributing to the ischemic brain injury. In preconditioning (PC), the exposure to a wide range of stressors activates inflammatory pathways and leads to upregulation of inflammatory molecules similarly to those induced by stroke. After the effects of the stressor have subsided, the inflammatory response is resolved during the early phase of PC and gene expression returns to basal levels. However, longer lasting epigenetic changes induced in the immune system components by micro-RNAs (miRNAs) and histone methylation (i.e., histone H3 trimethylation at lysine 4, H3K4me3) may reprogram inflammatory pathways to respond differently after an episode of severe ischemia, for instance favoring the expression of anti-inflammatory cytokines (IL-10, tumor growth factor-(TGF)-β and interferon-(IFN)-β that induces ischemic tolerance (IT).

Although post-ischemic inflammation is considered a major pathogenic factor in stroke, emerging evidence supports a beneficial role of selected inflammatory pathways. One such mechanism is the induction of endogenous neuroprotection through ischemic PC (Figure 3). The evidence summarized in this review indicates that the immune system plays a central role in the mechanisms eliciting neuroprotective responses after PC. While activation of IT-inducing immune pathways shows robust neuroprotection in animal models, the clinical translation of such approaches is complicated by side effects including systemic inflammation and immunodepression (Meisel et al., 2005; McColl et al., 2009). The identification of specific pathways and effector molecules involved in establishing inflammatory PC will help to design targeted PC protocols that can be potentially translated into clinical practice (Keep et al., 2010; Anrather and Hallenbeck, 2013; Narayanan et al., 2013). Notably, immune pathways are not only involved in PC induced by inflammatory mediators, but have also been implicated in several PC paradigms that are not triggered by inflammatory signals, such as ischemic and anesthetic PC, attesting a pivotal role for the immune system in mediating and/or establishing IT. Because of the involvement of inflammatory pathways in a wide variety of diseases, many of the immune components involved in PC are prime therapeutic targets and subjects of extensive drug development. Several proteins involved in PC, such as type I IFNs, are currently in clinical use, and re-purposing them for stroke therapy or prevention could provide a fast track for clinical translation. However, much remains to be learned. For example, few studies have investigated the role of specific immune cells in IT, an area of research that could result in cell-specific therapies and would therefore mitigate the likelihood of adverse effects associated with administration of inflammatory cytokines. In addition, a better understanding of the epigenetic mechanisms involved in IT could lead to therapies that preserve PC-induced epigenetic changes, thereby substantially extending the time frame in which IT is observed.

### Conflict of interest statement

The authors declare that the research was conducted in the absence of any commercial or financial relationships that could be construed as a potential conflict of interest.
